# PKCδ promotes the invasion and migration of colorectal cancer through c-myc/NDRG1 pathway

**DOI:** 10.3389/fonc.2023.1026561

**Published:** 2023-02-02

**Authors:** Hong-tao Jia, Yan-fei Shao, Xue-liang Zhou, Guang Yang, Ling Huang, Batuer Aikemu, Shu-chun Li, Cheng-sheng Ding, Xiao-dong Fan, Hi-ju Hong, Sen Zhang, Rui-jun Pan, Jing Sun

**Affiliations:** ^1^ Department of General Surgery, Ruijin Hospital, Shanghai Jiao Tong University School of Medicine, Shanghai, China; ^2^ Shanghai Minimally Invasive Surgery Center, Ruijin Hospital, Shanghai Jiao Tong University School of Medicine, Shanghai, China; ^3^ Shanghai Institute of Digestive Surgery, Ruijin Hospital, Shanghai Jiao Tong University School of Medicine, Shanghai, China

**Keywords:** colorectal cancer, protein kinase C δ, N-myc downstream-regulated gene 1, metastasis, epithelial-mesenchymal transition

## Abstract

**Objective:**

Colorectal cancer (CRC) is the third cause of expected cancer deaths both in men and women in the U.S. and the third most commonly diagnosed cancer in China Targeted therapy has been proven to improve overall survival for unresectable metastatic CRC. But the location of the primary tumor or the presence of various core driver gene mutations that confer resistance may limit the utility of targeted therapy. Therefore, it is of great significance to further elucidate novel mechanisms of invasion and metastasis of CRC and find potential novel therapeutic targets. Protein Kinase C Delta (PKCδ) plays an important role in various diseases, including tumors. In CRC, the function of PKCδ on proliferation and differentiation is mostly studied but various research results were reported. Therefore, the role of PKCδ in CRC needs to be further studied, especially in tumor invasion and metastasis in CRC which few studies have looked into.

**Methods:**

The expression of PRKCD was analyzed by the Genotype-Tissue Expression (GTEx) and The Cancer Genome Atlas (TCGA) databases and Immunohistochemical (IHC). Gene Ontology (GO), Kyoto Encyclopedia of Genes and Genomes (KEGG), and Gene Set Enrichment Analysis (GSEA) enrichment analysis were used to explore the biological functions and pathways related to PRKCD. Lentivirus transfection was used to construct CRC cell lines with overexpression and knock-down of PKCδ or N-myc Downstream Regulated Gene 1 (NDRG1). Cell invasion and migration assay, wound healing assay were used to detect the function of PKCδ and NDRG1 in the invasion and migration of cells. Flow cytometry analysis was used to detect the influence of PKCδ on the CRC cell cycles .Immunofluorescence histochemistry ,Immunoprecipitation Assay and qPCR were used to detect the relationship of PKCδ and NDRG1. Xenograft model was used to verify the role of PKCδ *in vivo*.

**Results:**

PKCδ is overexpressed in CRC and could promote Epithelial-Mesenchymal Transition (EMT) and the invasion and migration of CRC in vitro. We confirmed that PKCδ and the tumor suppressor factor NDRG1 had a co-localization relationship in CRC. PKCδ inhibited NDRG1 transcription and protein expression. Overexpressing NDRG1 could inhibit the function of PKCδ in promoting tumor invasion and migration. PKCδ could regulate c-Myc, one transcription factor of NDRG1, to down-regulate NDRG1. In vivo, overexpressing PKCδ could promote xenograft growth and volume. Thus, our results showed that PKCδ reduced the expression of NDRG1 through c-Myc, promoting the invasion and migration of CRC through promoting EMT.

**Conclusion:**

The increased expression of PKCδ in CRC tumor tissue could promote the invasion and migration of tumor cells, and one of the mechanisms may be regulating c-Myc to inhibit the expression of NDRG1 and promote EMT.

## Introduction

CRC is the third cause of expected cancer deaths both in men and women in the U.S. and the third most commonly diagnosed cancer in China (1), with an increasing incidence and mortality (2). Approximately 50-60% of patients diagnosed with CRC develop either lymph node or distant metastases (3). 70% to 75% of these mCRC patients survive beyond 1 year, 30% to 35% beyond 3 years, and fewer than 20% beyond 5 years from diagnosis (4). Targeted therapy has been proven to improve overall survival for unresectable mCRC. However, for patients with V-Ki-ras2 Kirsten rat sarcoma viral oncogene homolog or neuroblastoma RAS viral (v-ras) oncogene homolog sequence variations, neither anti- Epidermal Growth Factor Receptor nor anti-Vascular endothelial growth factor targeted therapy has fulfilled the optimal treatment goal (4). The location of the primary tumor or the presence of various core driver gene mutations that confer resistance may limit the utility of targeted therapy (5). Therefore, it is of great significance to further elucidate novel mechanisms of invasion and metastasis of CRC and find potential novel therapeutic targets.

PKCδ, encoded by *PRKCD*, is one of the PKC family members. PKC family has been intensely investigated in the context of cancer since the discovery as a receptor for the tumor-promoting phorbol esters (6). The PKC family contains different subtypes that have many targets and diverse cellular functions, including cell survival, proliferation, apoptosis, and migration, which are grouped into classical (α, β, γ), novel (δ, ϵ, η, θ), and atypical (ζ, λ, ι, μ) based on their structures and activation characteristics (7).

PKCδ generally slows proliferation, induces cell cycle arrest, and enhances the differentiation of various undifferentiated cell lines (8). In addition to normal physiological functions, PKCδ also plays an important role in various diseases, including tumors. The effect of PKCδ to stimulate growth has been generally identified in cancer, where it has been attributed to activation of the extracellular-signal-regulated kinase/mitogen-activated protein kinase cascade. But many evidence also reveal that activation of PKCδ induces apoptosis in prostate cancer cells and lung cancer cells (9).Therefore, the role of PKCδ in the tumor is contradictory, and the mechanism of its effect is still unclear. In CRC, the function of PKCδ on proliferation and differentiation is mostly studied but various research results were reported. PEP005 is a novel ingenol angelate that has antiproliferative effects by activating PKCδ in CRC (10). While PKCδ inhibitor Rottlerin inhibits cell division and proliferation of SW1116 cells (11). Therefore, the role of PKCδ in CRC needs to be further studied, especially in tumor invasion and metastasis in colorectal cancer which few studies have looked into.

In this study, we aimed to investigate the roles of PKCδ in tumor invasion and migration in CRC. Our results suggested that PKCδ, which activated c-Myc to decrease the expression of NDRG1, acted as an oncogene in CRC and played an important role in EMT process of CRC cells.

## Material and methods

### Cell culture and transfection

The RKO and HCT116 cell lines were purchased from the American Type Culture Collection, and both cell lines were authenticated b-y short tandem repeat profiling. All the cells were cultured in RPM-I-1640 medium (Gibco, Grand Island, NY, U-SA) supplemented with 10% fetal bovine serum (Gibco) and 1% penicillin-streptomycin (New Cell & Molecular Biotech, Suzhou, China) at 37 °C in 5% CO_2_. PKCδ overexpression and knockdown lenti-virus and their respective controls were purchased from Shanghai Genechem Co., LT-D. The sequence (5’-3’) of shRNA : PRKCD-RNAi1:Forward:ccggggCCGCTTTGAACTCTACCGTctcgagACGGTAGAGTTCAAAGCGGcctttttg,Reverse: aattcaaaaaggCCGCTTTGAACTCTACCGTCTCGAGACGGTAGAGTTCAAAGCGGCC;PRKCD-RNAi 2:Forward: ccgggcAGGGATTAAAGTGTGAAGActcgagTCTTCACACTTTAATCCCTgctttttg,Reverse: aattcaaaaagcAGGGATTAAAGTGTGAAGACTCGAGTCTTCACACTTTAATCCCTGC;PRKCD-RNAi3:Forward:ccggcaAGGCTACAAATGCAGGCAActcgagTTGCCTGCATTTGTAGCCTtgtttttg,Rev-erse: aattcaaaaacaAGGCTACAAATGCAGGCAACTCGAGTTGCCTGCATTTGTAGCCTTG. To construct the PRKCD stable overexpressed cell lines, we generated a lentiviral vector containing Ubi-MCS-SV40-Cherry-IRES-neomycin. The lentivirus plasmids were transfected with a multiplicity of infection of 50. 12 h after transfection, the medium was replaced. Stably knocked down and overexpression of PRKCD cells were selected with 0.4g/mL neomycin (Thermo Scientific) after infection. After 72 h, (Real-Time fluorescent quantitative Polymerase Chain Reaction) qPCR and immunoblots was used to assess the transfection efficiency. The infected cells were cultured with 0.2g/ml neomycin. Stable NDRG1 overexpression and knockdown cells were established as described previously (12).

### Tissue array

The paired tumor and normal colon samples were collected from 73 CRC patients from the Department of Gastrointestinal surgery of Ruijin Hospital, Shanghai Jiaotong University School of Medicine. The tissue array was made by Shanghai Runnerbio Biotechnology Co., Ltd. (Shanghai, China). The studies were reviewed and approved by the local ethics committee of Ruijin Hospital, Shanghai Jiao Tong University School of Medicine (No.2020-384).

### Immunohistochemical staining

Immunohistochemistry was carried out by Shanghai Runnerbio Biotechnology Co., Ltd. (Shanghai, China). Tissue Array were stained with primary antibodies against PKCδ (1:100, ab182126, Abcam). Scoring criteria of IHC: all sections were read by three pathologists under an ordinary light microscope and scored according to the German semi-quantitative scoring system. The specific scoring criteria is as follows: (1) staining intensity: 0: no staining; 1: mild staining; 2: moderate staining; 3: strong staining; (2) dying area: 0: < 10%; 1: 10%-25%; 2: 26%-50%; 3: 51%-75%; 4: > 76%. The IHC score of objective protein equals (1) × (2).

### Immunofluorescence assay

Sample sections (5μm) were deparaffinized and rehydrated before commencing the immunofluorescence assay. Slides were fixed with 4% paraformaldehyde and blocked for nonspecific binding using 10% goat serum and 5% FBS in PBS (Millipore) for 2 hours at room temperature. NDRG1 (ab124689, Abcam) or PKCδ (ab182126, Abcam) primary antibodies were incubated at 1:100 dilutions overnight at 4°C. A secondary antibody coupled to Alexa Fluor™ 488 or Cy3 was added to each slide for 1 hour at room temperature. Samples were mounted using a DAPI solution containing ProLong Gold antifade and mounting medium (Invitrogen). Fluorescence images were taken by an Olympus BX51 microscope equipped with a 40× lens objective (Olympus America Inc.). Images were captured with an Olympus DP-6 digital camera and processed with TissueFAXS Viewer.

### Cell invasion and migration assay

For invasion assay, the upper chamber was added with 50ul diluted Matrigel (the proportion of Matrigel: RPMI 1640 medium = 1:5). 10x10^4^ RKO cells and 7x10^4^ HCT116 cells were placed into the upper chamber of an insert (24-well insert, 8-μm pore size) (Corning Costar, Cambridge, MA, USA). The medium in the upper chamber did not contain serum, while the lower chamber contained 30% serum. For migration assay, 5x10^4^ RKO cells and 3x10^4^ HCT116 cells were placed into the upper chamber of an insert. The medium in the upper chamber did not contain serum, while the lower chamber contained 10% serum. After incubation for 24 h, the cells that did not invade through the upper membrane were removed with a cotton swab, whereas invading cells on the lower surface of the membrane were stained with 0.1% crystal violet (Solarbio Science & Technology, Beijing, China), and imaged using an inverted microscope (IX71; Olympus, Tokyo, Japan). Cell numbers were determined by counting the penetrating cells in five random fields per chamber, and the mean value was calculated. The wound-healing assay was used to evaluate the lateral migration ability of the cells. HCT116 and RKO cells (3-5 × 10^5^ cells/well) were seeded into 6-well plates, and 10 μL sterilization spear head was used to mark “one” line on the cells perpendicular to the culture plate after the cells were fully attached to the wall. Then the cells were washed three times by PBS and photographed under the microscope at 0 h and 24 h. Each experiment was done thrice and the cell migration ability was evaluated by the scratch healing rate: (Scratch area at 0 h - Scratch area at 24 h)/Scratch area at 0 h × 100%.

### Flow cytometry analysis

The transfected AS fibroblasts were harvested and fixed using 70% ethanol. Then the cells were washed with PBS and resuspended in binding buffer. Next, the cells were stained with propidium iodide (PI; Vazyme) for 20 min. The cell cycle distribution was analyzed by flow cytometry (BD Biosciences).

### Immunoblot assay

Whole cell proteins from each experimental group were extracted using RIPA lysis buffer (Thermo Scientific; Waltham, MA, USA) containing a protease inhibitor cocktail (Roche, Basel, Switzerland) according to the manufacturer’s instructions. Protein concentrations were determined using the Bicinchoninic Acid Kit (Sigma-Aldrich; St. Louis, MO, USA). Then, 50 μg of whole-cell proteins from each group were separated using 10% SDS-polyacrylamide gel electrophoresis and then blotted onto polyvinylidene difluoride filter membranes (Roche). The membranes were blocked in 5% nonfat milk for 1 h, washed three times with TBST at room temperature, and then incubated overnight at 4 °C with primary antibodies of NDRG1 (ab124689, Abcam), PKCδ (2058, Cell Signaling Technology), Claudin-1 (13255, Cell Signaling Technology); Vimentin (5741, Cell Signaling Technology), Slug (9585, Cell Signaling Technology), Snail (5741, Cell Signaling Technology), N-cadherin (13116, Cell Signaling Technology), E-Cadherin (14472, Cell Signaling Technology), GAPDH (51332, Cell Signaling Technology). After washing with TBST twice, membranes were incubated with anti-rabbit (SA00001-2, Proteintech) or anti-mouse (SA00001-1, Proteintech) secondary antibody (1:2000) at room temperature for 1 h. The blots were visualized with enhanced chemiluminescence reagent (Thermo Scientific) according to the manufacturer’s instructions.

### Immunoprecipitation assay

Cells were washed twice with PBS and lysed by RIPA. Protein was incubated with antibody PKCδ (2058, Cell Signaling Technology) or FLAG (MAB3118, Sigma-Aldrich) overnight at 4 s°C. This mixture was added to 30 ul of beads (Protein A/G PLUS-Agarose, sc-2003) from Santa Cruz Biotechnology (Santa Cruz, CA)and incubated for 4 h at 4°C. Then washed by PBS, resuspended in loading buffer, and incubated over 90°C for 10 min. The supernatant was separated on a 10% Bis-Tris gel. Then the mixture was detected by western blots.

### RNA extraction and qPCR

Total RNAs from cells were extracted using Trizol reagent (TaKaRa, Otsu, Japan) according to the manufacturer’s instructions. Following reverse transcription of isolated RNA (using the One Step PrimeScript Kit; TaKaRa; RR055A), the SYBR Premix Ex Taq kit (DRR420A, TaKaRa) was used to detect the expression of targeted genes and GAPDH using the CFX96 Real-Time PCR Detection System (Bio-Rad, Hercules, CA, USA). The cycle threshold (CT) value was calculated and the 2^−ΔΔCt^ method was used to quantify the relative amount of targeted genes, normalized with respect to GAPDH. The primer sequences: NDRG1 (5′-CTGCACCTGTTCATCAATGC-3′ and 5′AGAGAAGTGACGCTGGAACC-3′);PKCδ(5′-GTGCAGAAGAAGCCGACCAT-3′ and 5′-CCCGCATTAGCACAATCTGGA-3′);n-Myc(5′-ACCCGGACGAAGATGACTTCT-3′)and(5′-CAGCTCGTTCTCAAGCAGCAT-3′); c-Myc(5′-GGCTCCTGGCAAAAGGTCA-3′ and 5′- CTGCGTAGTTGTGCTGATGT-3′); Hypoxia inducible factor 1-alpha(HIF-1)5′- GAACGTCGAAAAGAAAAGTCTCG -3′ and 5′-CCTTATCAAGATGCGAACTCACA-3′); Activator protein-1(AP-1) (5′- AACAGGTGGCACAGCTTAAAC -3′ and 5′- CAACTGCTGCGTTAGCATGAG′); P53 (5′- CAGCACATGACGGAGGTTGT -3′ and 5′- TCATCCAAATACTCCACACGC-3′); GAPDH (5′-TTCAACAGCAACTCCCACTCTT-3′ and 5′TGGTCCAGGGTTTCTTACTCC-3′).

### Transfection of short interfering RNA

The c-Myc-siRNA was synthesized by Tsingke Biotechnology Co., Ltd.(Beijing, China). The sequences were as follows: Sense: 5’-GAACACACAACGUCUUGGATT-3’, Anti-sense: 5’-UCCAAGACGUUGUGUGUUCTT-3’. Transfections were performed using the Lipofectamine 3000 kit (Invitrogen; Thermo Fisher Scientific, Inc., Waltham, MA, USA) according to the manufacturer’s protocol. The knockdown-n efficiency was assessed by reverse transcription-quantitative polymerase chain reaction 48h after transfection.

### Xenograft model

Mice were cultivated under standard conditions following institutional guidelines. A total of 5 × 10^6^ RKO cells(PKCδ overexpression, knockdown and corresponding controls) were injected into male 4-week-old BALB/c nu/nu nude mice. Tumor size was measured every 7 days. Tumor volume(V) was determined by measuring the length and width of the tumor and using the formula V = (width*width*length)/2. 24 days after injection, all the mice were sacrificed and then tumor grafts were excised.

### Data acquisition

The *PRKCD* gene expression and related clinical data for bioinformatics analysis from the public data sets of Genotype-Tissue Expression (GTEx) and The Cancer Genome Atlas(TCGA) were downloaded from University of California, Santa Cruz (https://xenabrowser.net/datapages/). The GTEx database contains the transcriptional genome data of 42 healthy human tissues, including colorectal tissues, while the TCGA database covers the genomes and corresponding clinical data of various pan-cancer tissues.

### Functional analysis

In this study, Gene Ontology(GO), Kyoto Encyclopedia of Genes and Genomes(KEGG), and Gene Set Enrichment Analysis(GSEA) functional enrichment analyses were used to explore the downstream functions and pathways of PKCδ in CRC. GO enrichment analysis refers to annotated, comprehensive, and standardized vocabulary analysis for eukaryotic cells. It mainly includes three modules: biological process, molecular function, and cellular components. KEGG and GSEA enrichment analysis methods were used to analyze gene functions and pathways.

### Statistical analysis

IBM SPSS Statistical software (version 19.0) was used for statistical analysis. Differences were compared using a two-tailed Student t-test. Differences with a P value < 0.05 were considered statistically significant.

## Results

### PKCδ is overexpressed in multiple tumor tissues and related to tumorigenesis, metastasis, and poor prognosis in CRC

**Figure 1:**
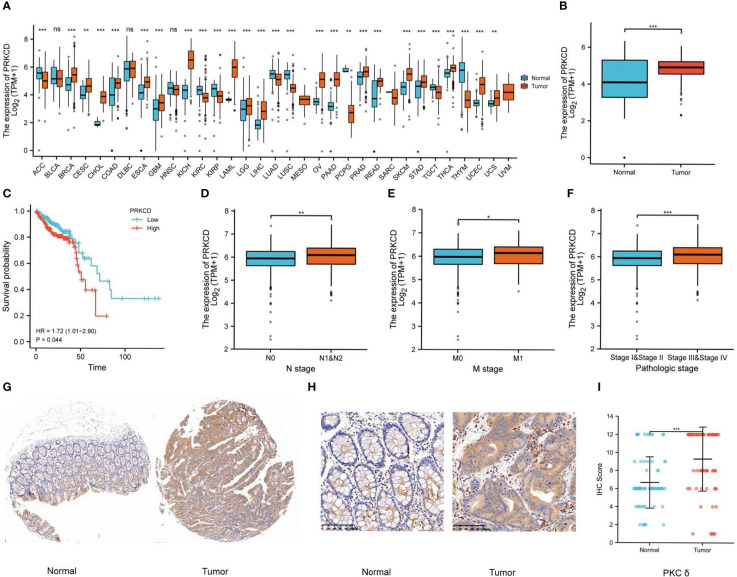
PKCδ is overexpressed in tumor tissues and related to poor prognosis in CRC. **(A)** The expression of PKCδ in pan-cancer and **(B)** CRC in the GTEx and TCGA database. The relationship between PKCδ and **(C)** the prognosis, **(D)** lymph node metastasis, **(E)** distant metastasis, and **(F)** tumor stage of CRC in the TCGA database. **(G-H)** The expression of PKCδ in CRC was detected by IHC. **(I)** Analysis of IHC results in 73 pairs of CRC tumor tissue and normal tissue by ggplot2. *p < 0.05, **p < 0.01, ***p < 0.001. ns, no significance.

First of all, we analyzed the expression of *PRKCD* in pan-cancer by using the GTEx and TCGA databases and found that in most solid tumors, the expression of *PRKCD* in tumor tissues was higher than that in normal tissues (**Figure 1A**). And in CRC tumor tissues(TCGA-COAD and TCGA-READ), the expression of *PRKCD* was also significantly higher than that in normal tissues (**Figure 1B**). Then, combined with the prognosis analysis of CRC patients in the TCGA database, we found that the expression level of *PRKCD* was related to the prognosis of CRC patients (HR=1.72, P=0.044). Patients with high expression of *PRKCD* had a poor prognosis (**Figure 1C**). In addition, combined with clinicopathological parameters, we also found that the expression level of *PRKCD* was also positively associated with lymph node metastasis, distant metastasis, and tumor stage (**Figures 1D–F**). Despite the pieces of evidence from the public database, a tissue array containing 73 pairs of CRC tumor tissues and matched normal tissues from our patient cohort was used to examine the expression of PKCδ by IHC (**Figures 1G, H**). The results showed that the expression of PKCδ in tumor samples was higher than that in normal samples, and the difference was statistically significant (P < 0.001), with the median IHC Score of PKCδ in the normal group is 6 (6-8) and in the tumor group is 12 (8-12), analyzed by the Wilcoxon rank-sum test (**Figure 1I**).

**Figure 2:**
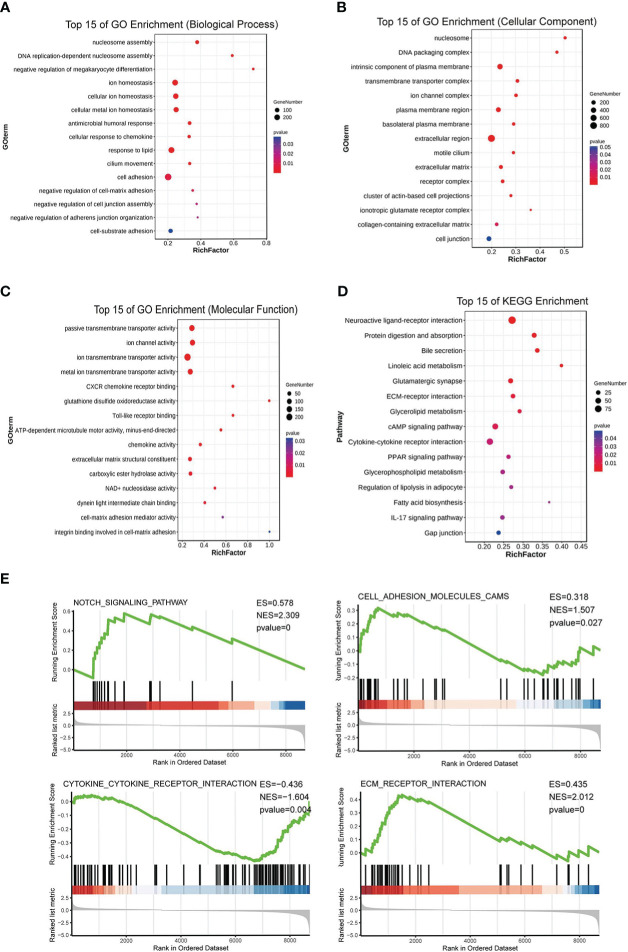
PKCδ is related to the tumorigenesis and metastasis of CRC. GO enrichment analysis showed that the functions of *PRKCD* was related to **(A)** ion channels, **(B)** cell junctions, and **(C)** cell adhesion. **(D, E)** KEGG and GSEA pathway analysis showed that *PRKCD* had a relationship with four core pathways related to tumor progression and metastasis, including Notch signal pathway, cell adhesion, ECM receptor interaction, and cytokine receptor interaction.

After the analysis above and the tissue array verification, we discovered that PKCδ had a significant role in CRC. Thus, the impact of PKCδ on tumorigenesis and metastasis of CRC was further explored. Using TCGA CRC data set with the median of *PRKCD* expression as the cut-off value, all CRC patients were divided into high expression group and low expression group. The differential gene patterns of the two groups were analyzed by GO, KEGG, and GSEA enrichment analysis to explore the biological functions and pathways related to *PRKCD*. The results of GO enrichment analysis showed that the functions of *PRKCD* related to ion channels, cell junctions, and cell adhesion (**Figures 2A–C**). At the same time, KEGG and GSEA pathway analysis showed that *PRKCD* had a relationship with four core pathways related to tumor progression and metastasis, including the Notch signal pathway, cell adhesion, ECM receptor interaction, and cytokine receptor interaction (**Figures 2D, E**). Based on the above results, we speculated that PKCδ may be related to the metastasis of CRC.

### PKCδ promotes the invasion and migration of CRC cells *in vitro*

**Figure 3:**
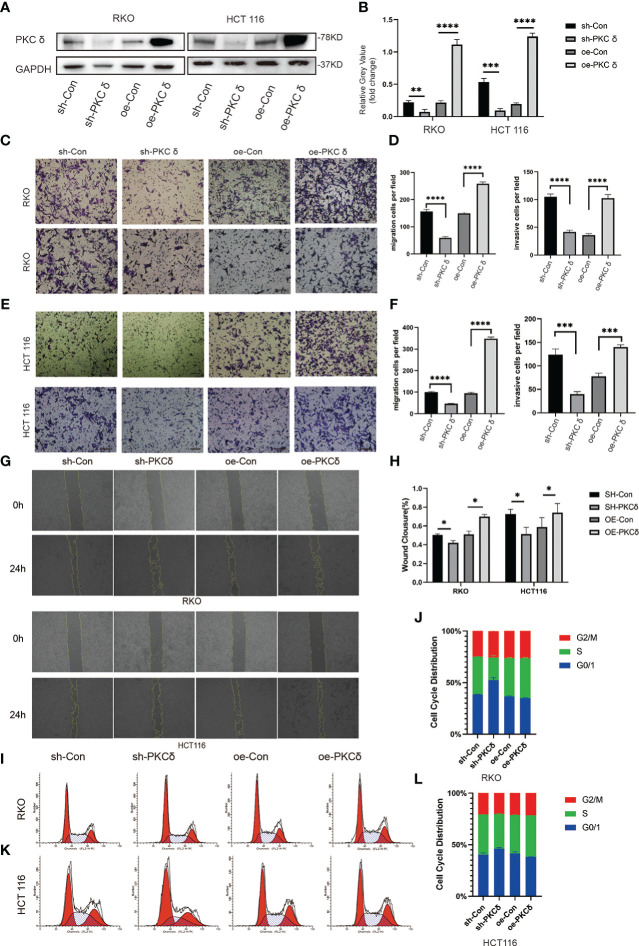
PKCδ promotes the invasion and migration of CRC cells **(A)** PKCδ overexpressed and knockdown and the corresponding negative control in RKO and HCT116 cells, determined by Western blots. **(B)** Columns were used to quantify the protein expression levels in **(A)** relative to GAPDH. **(C)** Cell migration and invasion assay to detect the effect of PKCδ on the invasion and migration of RKO cells. **(D)** Columns were used to quantify the migrated and invasive cells in **(C)**. **(E)** Cell invasion and migration assay to detect the effect of PKCδ on invasion and migration of HCT116 cells. **(F)** Columns were used to quantify the migrated and invasive cells in **(E)**. Wound-healing assay to the effect of PKCδ on the lateral migration ability of RKO and HCT116 cells **(G)**. **(H)** Columns were used to quantify the lateral migration cells in **(G)**. Flow cytometric analysis to detect the impact of PKC δ cell cycles of RKO **(I)** and HCT116 cells **(K)**. Columns were used to show the cell cycles of RKO **(J)** and HCT116 **(L)** cells.Scale bar. Scale bar: 20µm.*p < 0.05, **p < 0.01, ***p < 0.001, ****p < 0.0001.

To further prove that PKCδ promoted the invasion and migration of CRC cells, we used lentivirus transfection to construct CRC cell lines with overexpression and knock-down of PKCδ in both RKO and HCT116 cells. The expression levels of PKCδ were notably overexpressed and decreased after being transfected with overexpression and knock-down lentivirus compared with control groups, respectively (**Figures 3A, B**). The above results confirmed the transfection efficiency of PKCδ overexpression and knockdown. Next, cell invasion and migration assay showed that overexpression of PKCδ could enhance the invasion and migration ability while knocking down PKCδ inhibited the invasion and migration ability of RKO (**Figures 3C, D**) and HCT116 cells (**Figures 3E, F**). The wound-healing assay showed that overexpression of PKCδ could enhance the lateral migration ability while knocking down PKCδ inhibited the lateral migration ability of RKO and HCT116 cells (**Figures 3G, H**). According to the results of flow cytometric analysis, knocking down PKC δ could induce G0/G1 arrest in RKO and HCT116 cells, indicating that knocking down PKC δ played a key role in inhibiting proliferation of CRC cells (**Figures 3I–L**).

### PKCδ induces EMT of CRC cells *in vitro*

**Figure 4:**
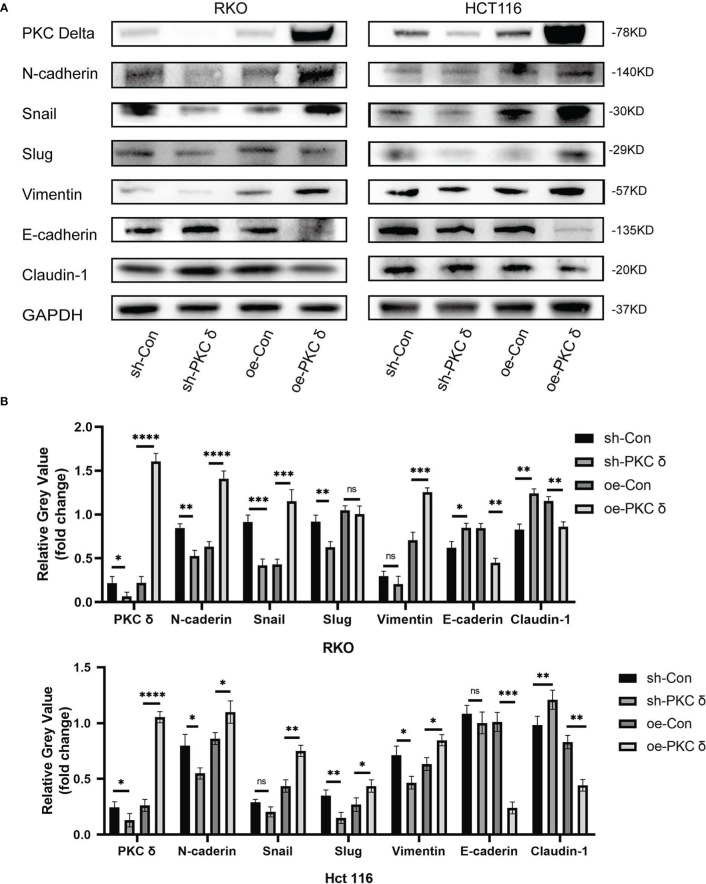
The overexpression of PKCδ induces EMT of CRC cells. **(A)** The different changes of EMT markers when overexpression and knocking down of PKCδ, detected by western blot in RKO and HCT116 cells. **(B)** Columns were used to quantify the protein expression in **(A)** relative to GAPDH. *p < 0.05, **p < 0.01, ***p < 0.001, ****p < 0.0001. ns, no significance.

After confirming that PKCδ can affect the invasion and migration ability of CRC cells, we further studied the underlying mechanism. Western blot results showed that overexpression of PKCδ downregulated the epithelial marker E-cadherin and Claudin-1, and upregulated the mesenchymal marker N-cadherin, Snail and Vimentin in RKO cells. Knocking down PKCδ upregulated the epithelial marker E-cadherin and Claudin-1, and downregulated the mesenchymal marker N-cadherin, Snail and Slug in RKO cells (**Figures 4A, B**). Meanwhile, western blot results showed that overexpression of PKCδ downregulated the epithelial marker E-cadherin and Claudin-1, and upregulated the mesenchymal marker N-cadherin, Vimentin, Snail and Slug in HCT116 cells. Knocking down PKCδ upregulated the epithelial marker Claudin-1, and downregulated the mesenchymal marker N-cadherin, Slug and Vimentin in HCT116 cells (**Figures 4A, B**). These results suggest that PKCδ could enhance the invasion and migration ability of CRC cells by promoting EMT.

### PKCδ and NDRG1 have a co-localization relationship in CRC

**Figure 5:**
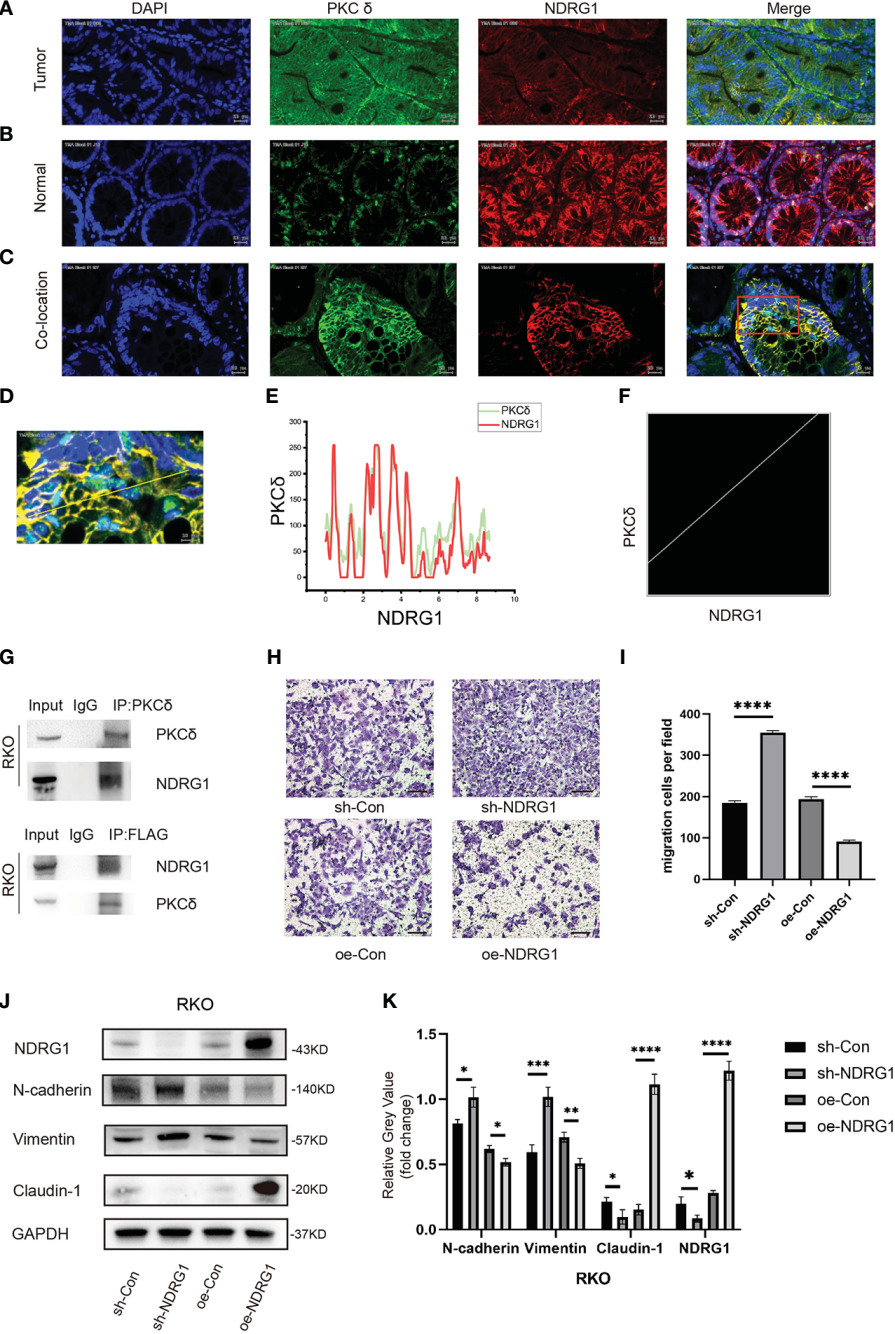
PKCδ and NDRG1 were co-located in CRC tissues. **(A)** The expression of PKCδ and NDRG1 in CRC tumor tissues, detected by immunofluorescence histochemistry. **(B)** The expression of PKCδ and NDRG1 in colorectal normal tissues, detected by immunofluorescence histochemistry. **(C)** The typical immunofluorescence images of PKCδ and NDRG1 in CRC tumor tissues for co-localization analysis. **(D)** Enlarged view of the image in the red box, which indicated the co-localization of PKCδ and NDRG1 in CRC, with the pixel acquisition area by the yellow line, detected by immunofluorescence histochemistry. **(E)** Pixel loci analysis of the expression of PKCδ and NDRG1 in colorectal cancer. **(F)** Analysis of the co-localization relationship of PKCδ and NDRG1 in CRC by software Fiji Image J, with the Pearson**’**s R-value 0.80, which indicated that PKCδ and NDRG1 were co-located strongly in CRC. **(G)** Co-immunoprecipitation was used to detect the relationship of PKCδ and NDRG1. **(H)** NDRG1 inhibited the migration of CRC cells by cell migration assay. **(I)** Columns were used to quantify the migrated and invasive cells in **(H)**. **(I)** Knocking down NDRG1 downregulated the epithelial marker Claudin-1 and upregulated the mesenchymal marker N-cadherin and Vimentin expression, while overexpression of NDRG1 showed the opposite trend in RKO cells, determined by Western blots. **(K)** Columns were used to quantify the protein expression in **(J)** relative to GAPDH. Scale bar. 20µm.*p < 0.05, **p < 0.01, ***p < 0.001, ****p < 0.0001.

Then, we studied the possible mechanism of PKCδ promoting EMT. Our research group has studied NDRG1 for a long time and found that the downstream mechanism of NDRG1 inhibiting colorectal cancer metastasis may involve multiple signaling pathways ^[14]^. But the upstream of NDRG1 lack further study. We reported that NDRG1 inhibited the EMT of CRC cells by regulating P21 and Caveolin-1^[15]^. In this study, we found that PKCδ promotes EMT to enhance the invasion and migration ability of CRC cells. Therefore, we assumed that there might be an interaction between the PKCδ and NDRG1. Immunofluorescence histochemistry results showed that PKCδ was overexpressed and NDRG1 was downregulated in CRC tumor tissues (**Figure 5A**). While in paired tissues, PKCδ was downregulated and NDRG1 was overexpressed (**Figure 5B**), indicating that PKCδ might have a negative relation with NDRG1. Then, immunofluorescence histochemistry results showed that PKCδ and NDRG1 were co-located in CRC tissues (**Figure 5C**) with the results of many overlaps between the two-pixel loci analysis (**Figures 5D, E**) and the Pearson’s R-value 0.80 (**Figure 5F**). Co-immunoprecipitation showed that PKCδ and NDRG1 have interaction in RKO cells (**Figure 5G**). Consistent with previous studies published by both our research team and others, cell invasion and migration assay showed that overexpression of NDRG1 could inhibit the invasion and migration of CRC cells (**Figures 5H, I**). Western blot results showed that knocking down NDRG1 downregulated the epithelial marker Claudin-1 and upregulated the mesenchymal marker N-cadherin and Vimentin expression, while overexpression of NDRG1 showed the opposite trend in RKO cells (**Figures 5J, K**). The above results showed that NDRG1 inhibited EMT and the invasion and migration of CRC cells.

### PKCδ inhibits NDRG1 transcription and protein expression

**Figure 6:**
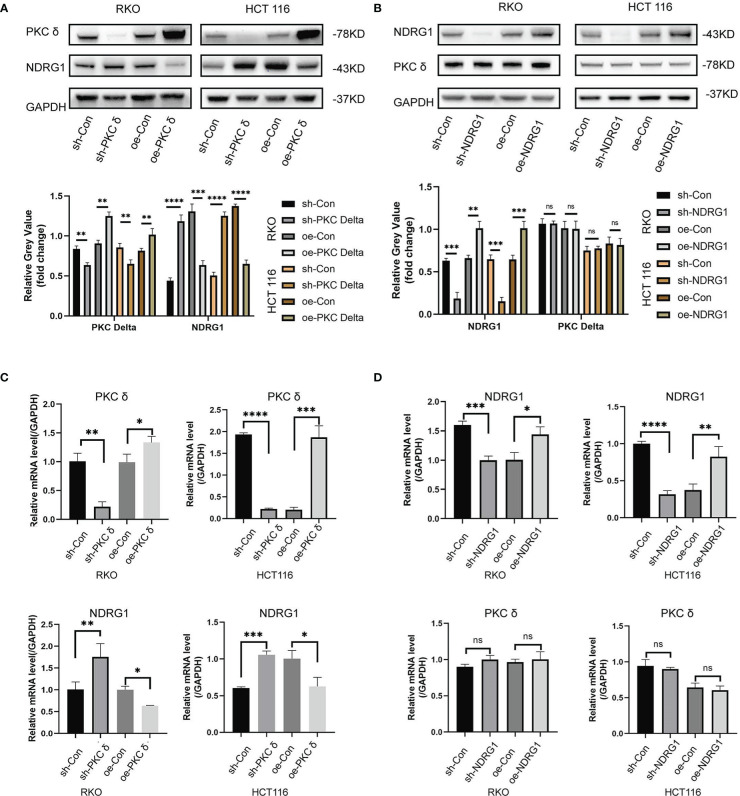
PKCδ inhibits NDRG1 transcription and protein expression **(A)** Western blot results showed that the expression of NDRG1 was significantly down-regulated when overexpressing PKCδ, and up-regulated when knocking-down PKCδ in RKO and HCT116 cells. **(B)** No significant change was found in the expression of PKCδ when NDRG1 overexpression or knocking down in RKO and HCT116 cells, determined by Western blots. **(C)** The level of NDRG1 was significantly down-regulated when overexpressing PKCδ, and up-regulated when knocking-down PKCδ in RKO and HCT116 cells, determined by qPCR. **(D)** No significant change was found in the level of PKCδ when NDRG1 overexpression or knocking-down in RKO and HCT116 cells, determined by qPCR. *p < 0.05, **p < 0.01, ***p < 0.001, ****p < 0.0001. ns, no significance.

Next, we further investigated the correlation between PKCδ and NDRG1 *in vitro*. Western blot results showed that the expression of NDRG1 was significantly downregulated in cells with PKCδ overexpression and the expression of NDRG1 was significantly upregulated in cells with PKCδ knocking down in RKO and HCT116 cells (**Figure 6A**), but there was no significant change in the expression of PKCδ after NDRG1 knocking down or overexpressing (**Figure 6B**). In addition, qPCR results also confirmed the above findings (**Figures 6C, D**). In conclusion, the expression of the NDRG1 in RKO and HCT116 cells was negatively regulated by PKCδ, and the expression of PKCδ was not affected by NDRG1.

**Figure 7:**
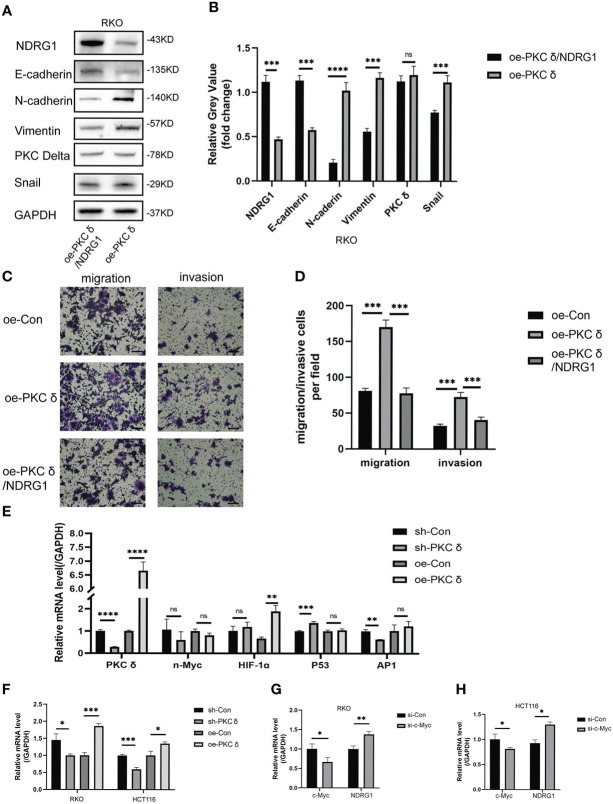
PKCδ down-regulated NDRG1 by promoting c-Myc expression. **(A)** Overexpressing PKCδ and NDRG1 downregulated the mesenchymal marker N-cadherin, Vimentin, and Snail and upregulated the epithelial marker E-cadherin in RKO cells, determined by Western blots. **(B)** Columns were used to quantify the protein expression levels in **(A)** relative to GAPDH. **(C)** PKCδ and NDRG1 overexpression inhibited the invasion and migration of RKO cells, detected by cell invasion and migration assay. **(D)** Columns were used to quantify the migrated and invasive cells in **(C)**. **(E)** The relationship between PKCδ and P53, AP-1, HIF-1α, or n-Myc in RKO cells, determined by qPCR. **(F)** The relationship between PKCδ and c-Myc in RKO and HCT116 cells, determined by qPCR. **(G) **The effect of PKCδ on the mRNA level of c-Myc in RKO cells, determined by qPCR. **(H)** The effect of PKCδ on the mRNA level of c-Myc in HCT116 cells, determined by qPCR. Scale bar: 20μm. *p < 0.05, **p < 0.01, ***p < 0.001, ****p < 0.0001. ns, no significance.

To further prove whether PKCδ promoted the invasion and migration of CRC cells by regulating NDRG1, we constructed stably transfected cells with co-overexpression of PKCδ and NDRG1. Western blot showed that the expression of NDRG1 in co-overexpression of PKCδ and NDRG1 cells was significantly higher than that in only PKCδ overexpressed cells (**Figures 7A, B**). After verifying the transfection effect, we demonstrated that comparing to the cells overexpressing PKCδ, the invasion and migration ability decreased in co-overexpression of PKCδ and NDRG1 colorectal cancer cells (**Figures 7C, D**). Meanwhile, co-expression of PKCδ and NDRG1 downregulated the mesenchymal marker N-cadherin, Vimentin, and Snail expression and upregulated the epithelial marker E-cadherin (**Figures 7A, B**), indicating that PKCδ may enhance the invasion and migration ability of CRC cells by inhibiting NDRG1 to promote EMT.

### PKCδ down-regulates NDRG1 by promoting c-Myc expression

Finally, we explored the possible mechanism of PKCδ inhibiting NDRG1. P53, AP-1, HIF-1α, n-Myc and c-Myc were transcription factors that directly or indirectly regulated the transcription of *PRKCD*. The qPCR results showed that there was no obvious positive or negative regulatory relationship between PKCδ and P53, AP-1, HIF-1α or n-Myc (**Figure 7E**). At the same time, it was found that the mRNA level of *c-Myc* was significantly up-regulated after overexpression of PKCδ, while significantly downregulated after knocking down PKCδ (**Figure 7F**). The qPCR assay verified that knocking down c-Myc up-regulated the mRNA level of *NDRG1* (**Figures 7G, H**), which indicated that c-Myc was a negative regulating factor of NDRG1. Therefore, it was concluded that PKCδ may inhibit the expression of NDRG1 in CRC cells by regulating c-Myc, promoting EMT, and enhancing the invasion and migration ability of CRC cells.

### PKCδ enhanced tumor growth *in vivo*

**Figure 8:**
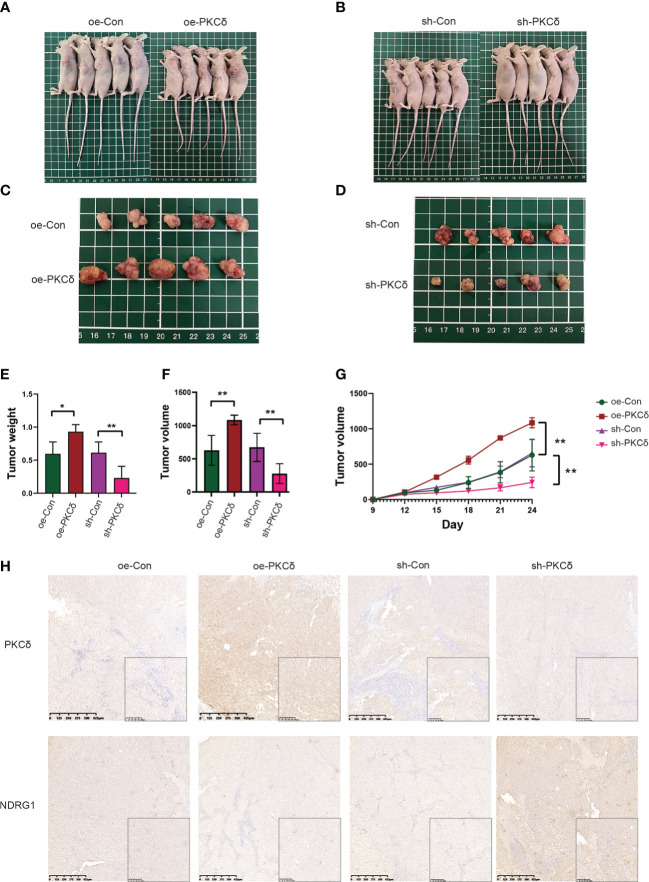
PKCδ enhanced tumor growth *in vivo*. Morphological observation of tumors formed after injection of nude mice with RKO cell lines that with PKCδ overexpression **(A, C)** or silencing **(B, D)**. **(E)** Average weight of xenografts. **(F)** Weight of tumors taken from nude mice after 24 days of growth. **(G)** Growth curves of tumors. **(H)** The representative IHC staining images of PKCδ and NDRG1 in tumor xenografts. *p < 0.05, **p < 0.01.

Based on the *in vitro* findings, we examined the effect of PKCδ on tumor growth in xenograft models. RKO cells with PKCδ overexpression, PKCδ knockdown and their control were injected into nude mice (**Figures 8A, B**). Not surprisingly, the tumor xenografts grew significantly bigger in the PKCδ overexpression group than those in the Vector-control group, while the xenografts grew significantly smaller in the PKCδ knockdown (**Figures 8C, D**). The average tumor weight (**Figure 8E**) and volume (**Figure 8F**) also showed similar trends. The volume growth rate of PKCδ overexpression group is faster, and that of PKCδ knockdown group is slower (**Figure 8G**). The expression of PKCδ and NDRG1 in xenograft tumors were further detected using IHC. NDRG1 expression was decreased in the PKCδ overexpression group and NDRG1 expression was increased in the PKCδ knockdown group, confirming that PKCδ negatively regulates NDRG1(**Figure 8H**).

## Discussion

PKC is the main intracellular receptor of tumor-promoting phorbol ester (13). The progression of therapeutics for targeting PKCδ or other PKC isoforms may have wide applications for the treatment of cancer and other diseases (14). PKCδ inhibitors were useful in inducing cell death in melanomas that had evolved resistance to v-raf murine sarcoma viral oncogene homolog B1 inhibitors, suggesting a potential clinical treatment strategy (15). In xenograft mouse models, anti-PKCδ therapy inhibited the activation of human insulin-like growth factor 1 receptor (16) In addition, inhibition of PKCδ can induce apoptosis. The xenograft model derived from pancreatic cancer patients also confirmed the effect of the inhibition of PKCδ (17). Therefore, it is of great significance to study the role of PKCδ in CRC, especially the invasion and migration.

In this study, we found that PKCδ was overexpressed in colorectal cancer. PKCδ was closely related to the poor prognosis. Then, through GO and KEGG enrichment analysis, it was found that PKCδ was closely related to cell junction, cell adhesion. Cell invasion and migration assay showed that the invasion and migration ability of overexpressed PKCδ colorectal cancer cells were significantly enhanced.

Epithelial-mesenchymal transition (EMT) is an important process in tumor invasion and metastasis (18). The decreased epithelial phenotype and increased mesenchymal phenotype enhanced the invasion and migration ability of tumors (19). In this study, western blot showed that overexpressed PKCδ downregulated the epithelial marker and upregulated the mesenchymal marker.

To confirm the relationship between PKCδ and NDRG1, we used the immunofluorescence histochemistry and found that they had a negative relationship in tissues and a co-localized relationship. Then, western blot and qPCR results showed that NDRG1 did not influence PKCδ, while PKCδ regulated NDRG1 negatively. Overexpressing NDRG1 rescued the enhanced EMT process of PKCδ, decreased the invasion and migration ability of RKO cells, upregulated the epithelial marker and downregulated the mesenchymal marker, which indicating that PKCδ could inhibit NDRG1 to promote EMT to enhance the invasion and migration ability of CRC cells.

To find the possible mechanism by which PKCδ regulated NDRG1, we started with the known transcriptional factors of NDRG1. Regulation was complex and involved interactions of NDRG1 and major metabolic regulators at the transcriptional, post-transcriptional and translational levels (20). It was found that inhibition of N-Myc/c-Myc could activate the promoter of NDRG1 and further enhance the expression of NDRG1 (21). In our study, the results of qPCR confirmed that c-Myc was a negative transcriptional regulator of NDRG1 and PKCδ regulated c-Myc negatively. Cancer stem cells (CSCs) have been proven to be essential for cancer metastasis. It has been reported that PKCδ is related to stemness, and inhibition of PKCδ can inhibit stemness of gastric cancer cells (22). Our group also found that NDRG1 inhibited stemness of CRC cells (23), so there may be a correlation between stemness and PKC δ/NDRG1 pathway, which will be researched in the next step.

In a word, we found that the increased expression of PKCδ in CRC tumor tissue could promote the invasion and migration of tumor cells, and one of the mechanisms may be regulating c-Myc to inhibit the expression of NDRG1 and promote EMT. New ideas and specific targets need to be further studied on PKCδ and verified by *in vivo* experiments.

## Data availability statement

The original contributions presented in the study are included in the article/supplementary material. Further inquiries can be directed to the corresponding authors.

## Ethics statement

The studies involving human participants were reviewed and approved by the local ethics committee of Ruijin Hospital, Shanghai Jiao Tong University School of Medicine (No.2020-384). The patients/participants provided their written informed consent to participate in this study.

## Author contributions

SZ, R-jP, and JS conceived and designed the experiments and provided financial support. H-tJ, Y-fS, X-lZ, GY, LH, and C-sD performed the experiments. Y-fS collected the bioinformatics data. BA, S-cL and X-dF collected the clinical sample. H-jH and SZ contributed to statistical analysis. H-tJ and JS interpreted the data and wrote the manuscript. SZ, R-jP, and JS revised the paper and provided final approval of paper. All authors discussed the results and commented on the paper. All authors contributed to the article and approved the submitted version.
